# Ballooning‐Interchange Instability in the Near‐Earth Plasma Sheet and Auroral Beads: Global Magnetospheric Modeling at the Limit of the MHD Approximation

**DOI:** 10.1029/2020GL088227

**Published:** 2020-07-14

**Authors:** K. A. Sorathia, V. G. Merkin, E. V. Panov, B. Zhang, J. G. Lyon, J. Garretson, A. Y. Ukhorskiy, S. Ohtani, M. Sitnov, M. Wiltberger

**Affiliations:** ^1^ The Johns Hopkins University Applied Physics Laboratory Laurel MD USA; ^2^ Space Research Institute Austrian Academy of Sciences Graz Austria; ^3^ Department of Earth Sciences University of Hong Kong Hong Kong; ^4^ Department of Physics and Astronomy Dartmouth College Hanover NH USA; ^5^ High Altitude Observatory, National Center for Atmospheric Research Boulder CO USA

**Keywords:** GAMERA, ballooning‐interchange, auroral beads

## Abstract

Explosive magnetotail activity has long been understood in the context of its auroral manifestations. While global models have been used to interpret and understand many magnetospheric processes, the temporal and spatial scales of some auroral forms have been inaccessible to global modeling creating a gulf between observational and theoretical studies of these phenomena. We present here an important step toward bridging this gulf using a newly developed global magnetosphere‐ionosphere model with resolution capturing ≲ 30 km azimuthal scales in the auroral zone. In a global magnetohydrodynamic (MHD) simulation of the growth phase of a synthetic substorm, we find the self‐consistent formation and destabilization of localized magnetic field minima in the near‐Earth magnetotail. We demonstrate that this destabilization is due to ballooning‐interchange instability which drives earthward entropy bubbles with embedded magnetic fronts. Finally, we show that these bubbles create localized field‐aligned current structures that manifest in the ionosphere with properties matching observed auroral beads.

## Key Points

- We present the first global magnetosphere simulation to reveal ballooning‐interchange instability of a narrow *B*_*z*_ minimum in the near‐Earth magnetotail
- The instability is prominent during the substorm growth phase and generates earthward entropy bubbles with embedded magnetic fronts
- The bubbles drive mesoscale ionospheric field‐aligned currents and auroral structures (beads) with properties matching to those observed

## 1 Introduction

The existence of a minimum of the equatorial northward magnetic field component, *B*_*z*_, in the Earth's magnetotail has been suggested theoretically as a feature of steady adiabatic convection (e.g., Hau, 1991). Such a minimum forms as plasma sheet convection proceeds and the magnetic field of the inner tail becomes progressively more stretched (e.g., Wolf et al., 2009). The presence of a *B*_*z*_ minimum implies a tailward *B*_*z*_ gradient further from the Earth, and such gradients, in turn, can be unstable to a number of plasma instabilities, including tearing (Sitnov & Schindler, 2010) or its magnetohydrodynamic (MHD) analog (Birn et al., 2018; Merkin et al., 2015; Merkin & Sitnov, 2016), flapping (Erkaev et al., 2008), or ballooning/interchange (BI) (e.g., Pritchett & Coroniti, 2011). Because of their important role in the plasma sheet dynamics, *B*_*z*_ minima and tailward gradients have received significant interest recently (see Sitnov, Birn, et al., 2019, for a review).

Measuring magnetic gradients in situ is notoriously difficult, but low values of the magnetic field are indeed occasionally observed in the near‐Earth tail, particularly in the transition region from a dipolar to a tail‐like configuration (9–12 *R*_E_ from Earth), suggesting the presence of a *B*_*z*_ minimum (Ohtani & Motoba, 2017). Panov and Pritchett (2018) used a fortuitous alignment of five Time History of Events and Macroscale Interactions during Substorms (THEMIS) probes to suggest that such regions may persist for multiple hours, be azimuthally extended (up to 10 *R*_E_) and radially localized (within 2.5 *R*_E_), and exhibit signatures of BI instability (“interchange heads”). Sergeev et al. (2018) inferred a *B*_*z*_ minimum in the near‐Earth tail from observations of isotropic energetic electrons at low altitudes equatorward of an anisotropic precipitation region, suggesting a magnetic field enhancement further tailward. Large‐scale tailward *B*_*z*_ gradients have also been detected in data mining‐based reconstructions of the geomagnetic field during substorms (Stephens et al., 2019; Sitnov, Stephens, et al., 2019).

In this Letter we demonstrate the formation of a radially narrow ( ≲ 0.5 *R*_E_) *B*_*z*_ minimum, and the corresponding local flux tube entropy depletion, in the near‐Earth magnetotail during a substorm growth phase. Such a spatially localized feature would be particularly difficult to detect using any of the above data‐based techniques. Instead, we employ our newly developed global magnetosphere magnetohydrodynamic (MHD) model, Grid Agnostic MHD for Extended Research Applications (GAMERA). A distinguishing feature of the model (see section 2) is its high resolving power, enabled by sophisticated numerical algorithms (Zhang et al., 2019), which allowed us to not only reveal the *B*_*z*_ minimum but also demonstrate its dynamic nature due to the BI motion of magnetic flux tubes. These dynamics result in the generation of localized perturbations in ionospheric field‐aligned currents and corresponding auroral signatures commonly referred to as beads (e.g., Henderson, 2009; Motoba et al., 2012; Nishimura et al., 2016). A link between the interchange heads in the plasma sheet (or entropy bubbles, since they are depleted of flux tube entropy) and localized auroral structures (beads) has been demonstrated recently using fully kinetic simulations (Panov et al., 2019). Here, we present an independent corroboration of this connection, with the difference that both the plasma sheet and the ionosphere are included as part of our global simulation, which encompasses the entirety of solar wind‐magnetosphere‐ionosphere interaction and thus avoids complications resulting from artificially imposed boundary conditions in regional magnetotail models.

## 2 Model Description and Simulation Setup

The results presented here are the first published application of our newly developed MHD model, GAMERA, to simulation of Earth's magnetosphere‐ionosphere system. GAMERA was designed to be a successor to the Lyon‐Fedder‐Mobarry (LFM) model (Lyon, Fedder, et al., 2004), which has been at the forefront of space physics modeling since its initial development in the early 1980s (Lyon, Brecht, et al., 1980). The GAMERA development has been guided by the goal of preserving the core numerical philosophy of LFM, characterized by high‐order spatial reconstruction, aggressive flux‐limiting, and intrinsically divergence‐free magnetic field updates on arbitrary hexahedral grids, while modernizing and improving the algorithms and implementation. The numerical details of the core MHD algorithm, as well as the results of a suite of standard MHD test problems, have been recently presented in exhaustive detail by Zhang et al. (2019). The extension of the MHD kernel to a full magnetospheric model requires numerous additional modules, for example, electrostatic coupling to the ionosphere, ionospheric conductance models, imposition of the upstream solar wind boundary condition, and treatment of the geomagnetic background field. In this regard, our approach is broadly similar to LFM (Lyon, Fedder, et al., 2004; Merkin & Lyon, 2010), and will be described in more detail elsewhere.

To focus on the basic physical processes that occur during a substorm growth phase, we follow the long heritage of synthetic substorm simulations using the LFM model (Fedder & Lyon, 1987; Gordeev et al., 2017; Lyon et al., 1981; Slinker et al., 1995). At the same time, we leverage the resolving power of our algorithms and GAMERA's ability to efficiently use the available computational resources to simulate a global magnetosphere at a resolution approaching the ion kinetic scale. The inner boundary condition of the simulation is imposed on a spherical surface of radius 2 *R*_E_ centered on Earth and assumes current closure through a thin‐shell ionosphere at the altitude of ∼ 120 km (Merkin & Lyon, 2010). In this simulation a constant Pedersen conductance of Σ_*p*_ = 10 S was used and no Hall conductance. The magnetosphere is preconditioned using a period of alternating southward and northward interplanetary magnetic field (IMF) orientations for a period of 8 hr at which point the IMF is again turned southward (*B*_*z*_ = −5 nT) and is held fixed for the remainder of the simulation: *n* = 5 cm^−3^ and *V*_*x*_ = −400 km/s. We use *T* = 0 to refer to the time of the final southward IMF turning, that is, the beginning of the growth phase.

**Figure 1:**
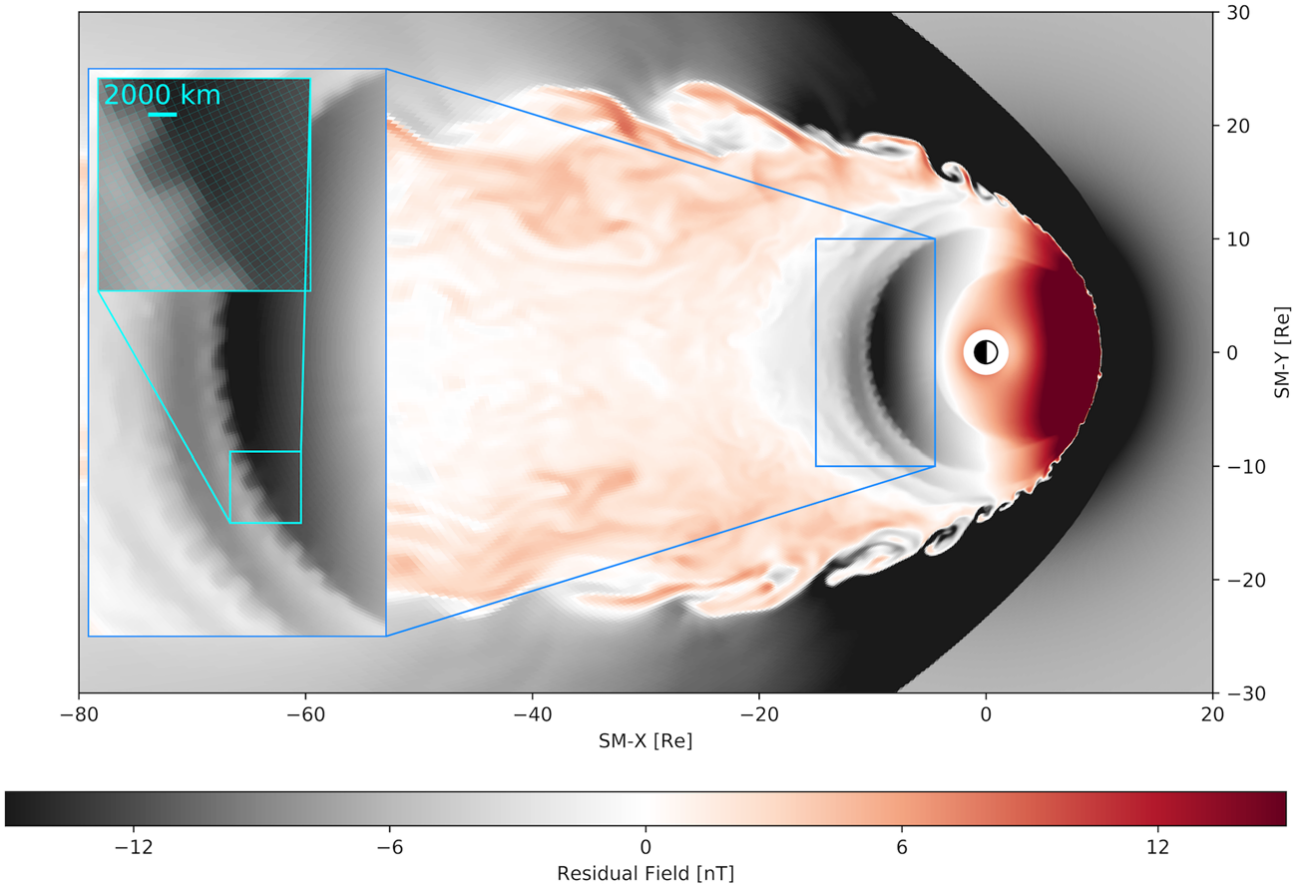
A snapshot of the simulation at *T*=+30 min depicts the residual (with the dipole subtracted) SM *B*_*z*_ magnetic field in the equatorial plane. Progressive zoom‐ins on the BI structure are shown. The deepest layer of zoom demonstrates the actual grid resolution and how it relates to the BI heads.

This simulation uses a grid with approximately 2 times the number of cells in each dimension relative to the highest resolution LFM simulation ever performed (“OCT”) (Merkin, Lyon, et al., 2013; Merkin, Anderson, et al. 2013; Merkin et al., 2019; Wiltberger et al., 2015). Specifically, the grid uses 382×512×382 cells in the radial, polar, and azimuthal directions (with respect to the Solar Magnetic, SM, *X* axis). The distorted nature of the grid allows us to concentrate cells in regions of interest, for example, the plasma sheet, magnetopause and bow shock, while smoothly transitioning to coarser regions. In the central plasma sheet (near *X*_SM_ ≈ −20 *R*_E_) the characteristic grid lengthscale, $\ell =\sqrt[{3}]{\Delta V}$ where Δ*V* is the cell volume, is *ℓ* ≈ 500 km and ≲ 30 km azimuthal resolution in the auroral ionosphere (0.5° × 0.5°). Most critical, however, is Δ*z* in the central plasma sheet which is 300 km, comparable to the ion kinetic scale in this region. Furthermore, the high‐order spatial reconstruction allows us to capture physical features essentially at the grid scale with fewer cells than lower‐order schemes would (Zhang et al., 2019). Figure 1 shows a snapshot from the simulation and, in the deepest layer of zoom, the actual equatorial grid used and how it compares to the BI heads discussed below. A similar three‐dimensional visualization is shown in Figure S1 in the supporting information (SI).

## 3 Results

### 3.1 Flux Redistribution During Growth Phase

**Figure 2:**
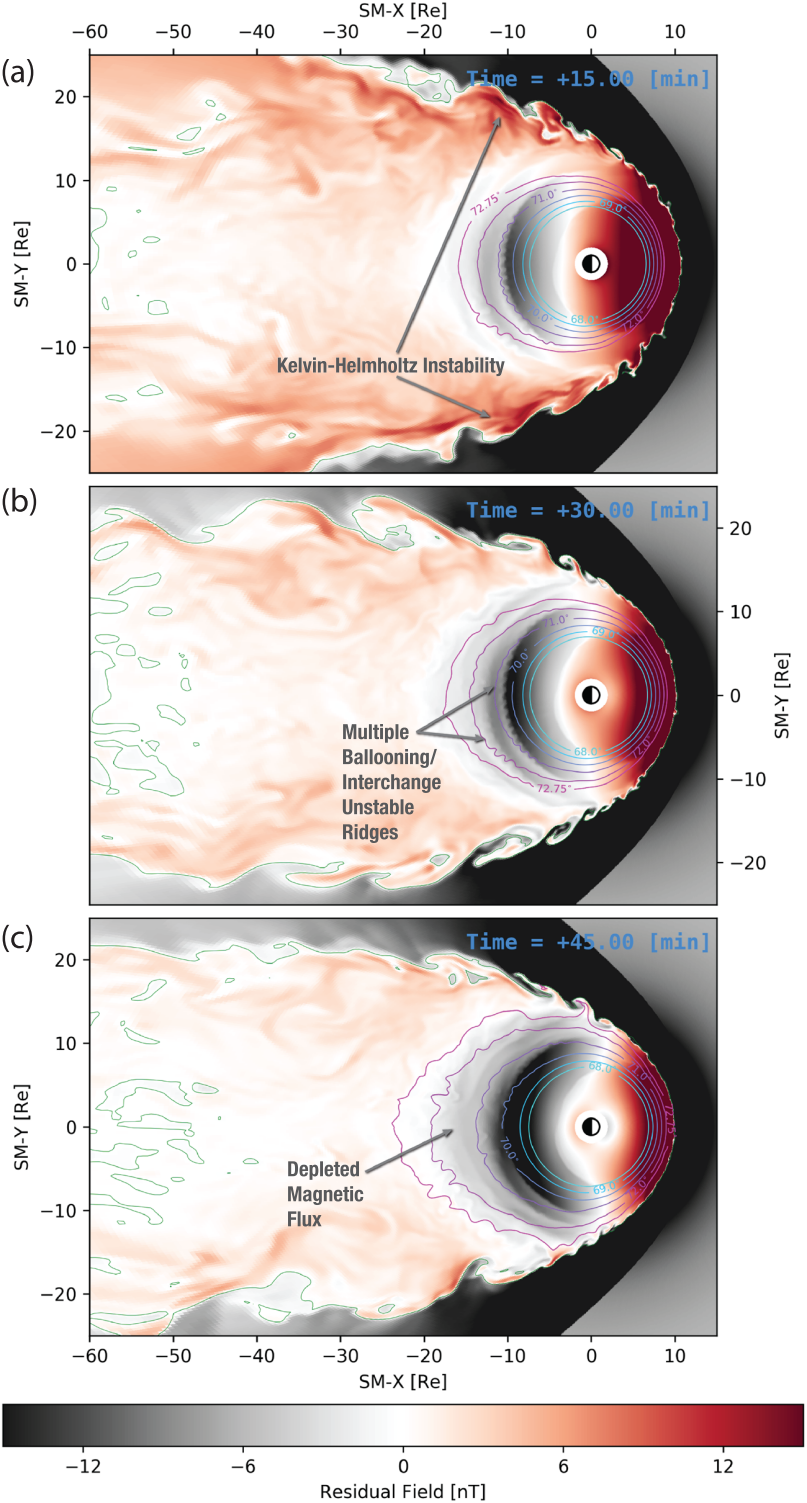
Simulation at a glance. (a–c) Snapshots of the simulation at 15 min increments after the southward IMF turning. Each panel depicts the equatorial residual, that is, deviation from dipolar, northward magnetic field and contours of *B*_*Z*_=0 (green). Also shown are contours of constant magnetic latitude mapped to the equatorial magnetosphere (cyan‐magenta).

Figure 2 shows an overview of the simulation. Each panel depicts the residual (with the dipole subtracted) SM *B*_*z*_ magnetic field in the equatorial plane for the different times elapsed since the IMF southward turning. We first point out the high resolving power of the simulation manifested in the fine structure captured, for instance, at the magnetopause boundary due to the Kelvin‐Helmholtz instability (KHI) (cf. Merkin, Lyon, et al. 2013; Sorathia et al., 2019). Figure 2a shows the simulation 15 min after the southward turning. At this time, the IMF discontinuity has already propagated down tail so it is not apparent in the figure, but the night side of the magnetosphere is still largely a reflection of its state prior to the turning. Magnetic flux has been accumulated during the preceding period of northward IMF and stored in the central plasma sheet and at the flanks, indicated by the positive residual fields, shades of red, in those regions.

Figure 2b depicts the state of the simulation 30 min into the growth phase. At this time, the stretching of the geomagnetic field in the near‐Earth magnetotail is evident relative to the previous snapshot (Figure 2a) as well as to the beginning of the growth phase (see Figure S2 in SI). The stretching can be seen by observing the contours of constant magnetic latitude projected to the equator shown in all panels of Figure 2. It is also apparent from the more depleted magnetic field represented by the darker shades of gray color in the nightside magnetosphere. This magnetic flux depletion is a known feature of the growth phase (e.g., Sitnov, Birn, et al. 2019) which occurs due to the flux evacuation by convection to the flanks and the dayside (Gordeev et al., 2017; Hsieh & Otto, 2014; Merkin et al., 2019). Finally, Figure 2c demonstrates further depletion of the magnetic flux at the end of the growth phase. In particular, the purple contour of 72.75° magnetic latitude has moved beyond 20 *R*_E_ at midnight.

Now we draw the reader's attention to the formation of strong undulations in the magnetic field distribution on the night side due to the BI instability seen in Figure 2 and highlighted in Figure 1. These structures will be the focus of the remainder of this paper. We note here that their counterparts in the ionosphere are similar undulations in the distribution of the auroral field‐aligned currents, which we return to in section 3.3. It is also worth noting that in addition to the primary, namely the inner most in Figure 1, ridge of BI “ripples” the simulation exhibits additional ridges, less pronounced and with a tendency to be more variable, up until the region is disrupted by fast flows driven by downtail reconnection. One of these ridges is clearly evident in Figure 2b, and these structures can be seen in dynamics in Movie S1 in SI. All of these ridges correspond to the tailward walls of local magnetic field minima (i.e., tailward *B*_*z*_ gradients) which are discussed in more detail below.

### 3.2 Ballooning‐Interchange Instability in the Magnetosphere

Returning to Figure 1 for a moment, the inset in the upper left indicates that the azimuthal scale size of the residual *B*_*z*_ ripples is on the order of ∼ 4,000 km, and is well resolved by the simulation. This is consistent with the observed cross‐tail scales of the BI waves, corresponding to their half period, estimated to lie between 4,500 and 6,000 km (Panov et al., 2012; Panov & Pritchett, 2018). Comparison of the auroral manifestations of these magnetospheric structures with observations is presented in section 3.3.

**Figure 3:**
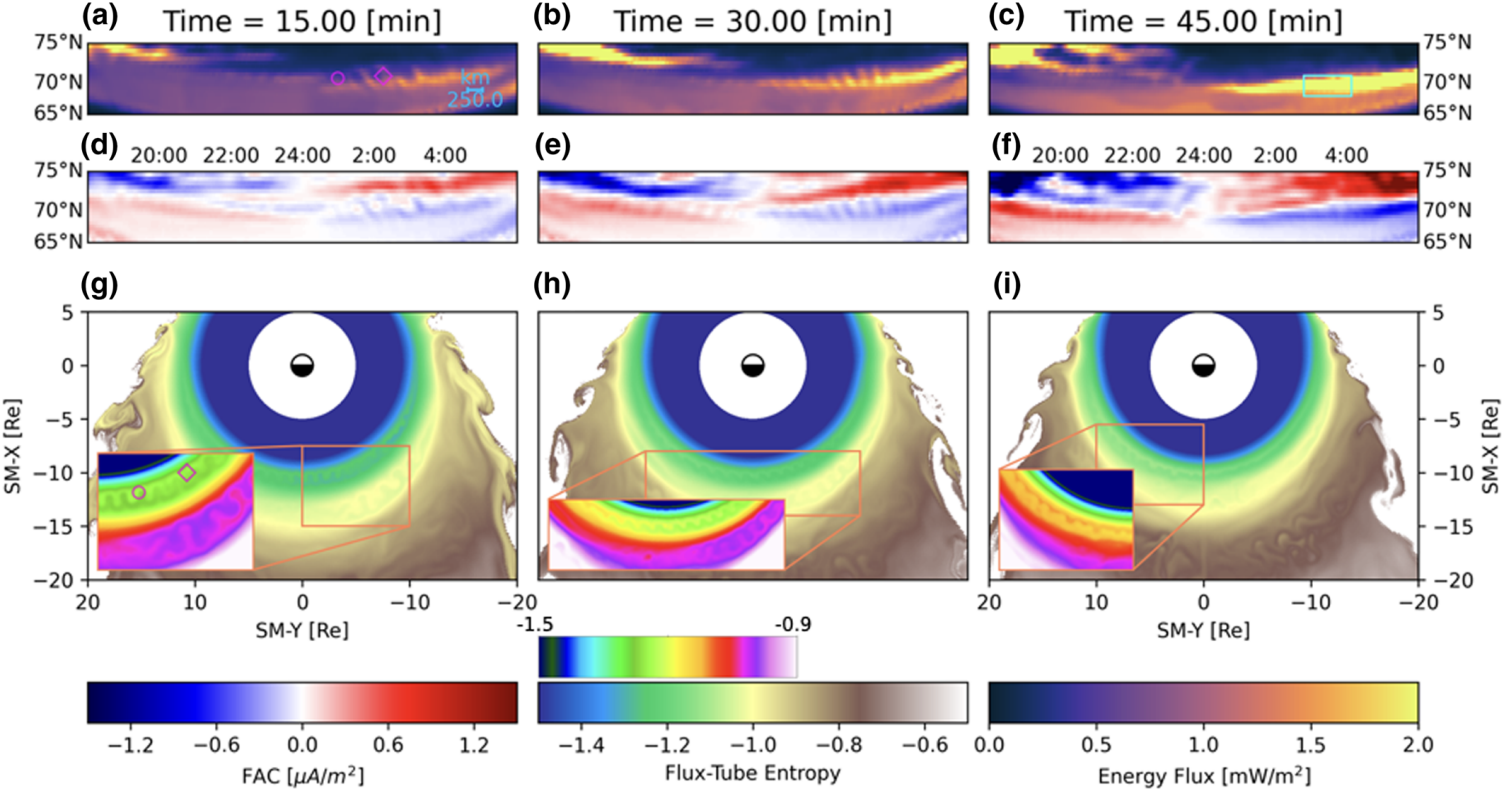
Ionospheric consequences of BI instability in the magnetosphere. Snapshots of the simulation at 15 min increments after the southward IMF turning are shown. (a–c) Predicted precipitating electron energy flux. (d–f) Ionospheric field‐aligned current density (downward positive). (g–i) Flux tube entropy with inset boxes zoomed in on BI instability heads with the former using the bottom colormap with larger dynamic range than the inset boxes. The magenta glyphs in (a) and (g) depict points in the equator and ionosphere connected by magnetic field lines. The cyan box in (c) is the region depicted more closely in Figure 4.

Furthermore, Figure S2 indicates that the residual *B*_*z*_ ripples correspond with a localized minimum of the total equatorial magnetic field *B*_*z*_, while Figures 3g–3i and Figure S3 in SI demonstrate that this (and other) *B*_*z*_ minima are associated with the corresponding reversed (earthward) flux tube entropy gradients. To be more precise, it is the tailward *B*_*z*_ gradient that corresponds to an earthward entropy gradient (c.f.  Birn et al., 2018, Figures 4e,f) and thus it is these regions that become BI unstable (Bernstein et al., 1958). Here, by flux tube entropy we understand the standard expression (e.g., Birn et al., 2009), $S=\int p^{1/\gamma }dl/B$, where *p* is the plasma pressure, *γ* is the polytropic index, *B* is the magnetic field magnitude, and integration is performed along the flux tube from one ionospheric foot point to the other. The fact that the undulations in the equatorial *B*_*z*_ distribution (as well as their apparently unstable dynamics evident from Movies S1 and S2) occur in regions of inverted flux tube entropy gradients is an explicit demonstration of BI instability of these regions. Signatures of cross‐tail structure expected of ballooning instability have also been reported in global simulations by Raeder et al. (2010). However, to our knowledge, the present paper is the first explicit demonstration of BI instability of localized equatorial *B*_*z*_ minima in a global magnetosphere simulation of a substorm growth phase according to the original energy principle criterion (Bernstein et al., 1958).

### 3.3 Auroral Manifestation of BI Instability

Figure 3 further concentrates on the equatorial distribution of the flux tube entropy (panels g–i) as well as the corresponding ionospheric distributions of field‐aligned currents (panels d–f) and precipitating electron energy flux (panels a–c). The figure depicts the state of the simulation at three different moments of time during the growth phase, 15 min apart. The electron energy flux (Figures 3a–3c) is derived from the MHD pressure, density and field‐aligned currents at the inner boundary of the simulation (2 *R*_E_) using the model by Fedder et al. (1995) (see also Wiltberger et al., 2009; Zhang et al., 2015), and it includes both diffuse and discrete precipitation contributions. The latter is modulated by the field‐aligned currents via the Knight relation (Knight, 1973), and thus we observe significantly elevated electron fluxes in regions of upward (negative) current densities. We stress that the currents shown in Figure 3 are computed near the inner boundary of the simulation and largely retain the spatial structure of their magnetospheric driver (Figures 3g–3i). This is enabled by the high resolving power of our simulation (see Raeder et al., 2012 for discussion of magnetosphere‐ionosphere mapping issues for such fine auroral structures.)

In the ionosphere, we point out the relatively narrow sheet of upward current at premidnight. This result is consistent with the simulations of the quiet growth arc using the Rice Convection Model by Yang et al. (2013). Even given the high resolution of our global simulation, the thickness of this current sheet is ∼ 1° in latitude which corresponds to ∼ 113 km at a 100 km altitude. This is thicker than the arc in the two‐dimensional simulations by Yang et al. (2013), which afford a much higher latitudinal resolution. Still, given the morphological consistency of these upward current sheets in the two simulations, we hypothesize that we are seeing a prototypical quiet growth phase arc in our global simulation.

Figures 3g–3i demonstrate the existence of entropy bubbles at different times during the growth phase and that they are distributed in MLT, appearing in the evening, midnight, and morning sectors. Correspondingly, both R1 and R2 field‐aligned currents (Figures 3d–3f) exhibit pronounced undulations throughout the night side auroral ionosphere. Movie S2 demonstrates the dynamics of this picture and additionally indicates the equatorial mapping of lines of constant latitude in the ionosphere to better match ionospheric and magnetospheric features of the simulation. The undulations in the field‐aligned currents correspond to bead‐like features in the discrete electron precipitation (Figures 3a–3c), which delineate localized upward currents. It is worth noting that the auroral beads in our simulation appear both in R1 and R2 current regions throughout the growth phase and thus are not necessarily onset arc features. In observations, auroral beads have also been reported both as onset arc structures (Henderson, 2009; Kalmoni et al., 2015, 2017; Nishimura et al., 2016) and not (Panov et al., 2019; Uritsky et al., 2009; Xing et al., 2020).

**Figure 4:**
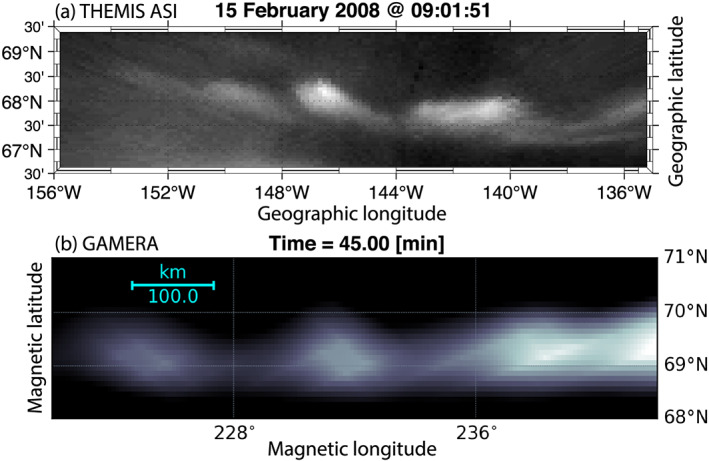
(a) Miller projection of the THEMIS All‐Sky Imager (ASI) at Fort Yukon (located at 66.56°N, 145.214°W) on 15 February 2008 at 09:01:51 UT. (b) A zoom‐in on a region of Figure 3c (cyan box) reprocessed using a different color scale to facilitate the comparison with panel (a).

For a visual comparison of the simulated beads with auroral observations, Figure 4a shows Miller projection of auroral observations from the THEMIS All‐Sky Imager (ASI) in Fort Yukon on 15 February 2008 at 09:01:51 UT. The imager was located at 66.56°N, 145.214°W (i.e., near the middle of the bottom axis). The auroral beads were detected between about 09:01:00 and 09:02:30 UT, appeared to be about 2° wide and also separated by about 2°. The beads propagated westward at a velocity of about 0.2° per second. Figure 4b shows the results of our simulation zooming in on a region of Figure 3c spanning exactly the same distance in both longitude and latitude as Figure 4a. Even given the high resolution of our simulation, the beads in Figure 4b still appear underresolved compared to the observations, although their salient features like the size and separation are very similar to those observed. While the observations in Figure 4a are taken in the evening sector and the beads propagate westward, the beads in the simulation are more pronounced in the morning sector R2 current branch (Figures 3a–3c). This is because this auroral region both maps to the BI unstable region in the magnetosphere and has the field‐aligned current of the right sense (upward) to produce discrete electron precipitation (see Movie S2 for more explicit mapping demonstration.) Still, Figures 3c, 3f, and 3i exhibit bead‐like perturbations of the thin R1‐sense current sheet in the evening sector, which we interpreted above as the analog of the growth phase arc discussed in the context of RCM simulations by Yang et al. (2013).

## 4 Discussion and Conclusions

Figure S2 demonstrates that the narrow *B*_*z*_ minimum appears in the simulation even prior to the beginning of the growth phase (*T* = 0 min). Thus, it is a persistent feature of the simulated magnetosphere, including during northward IMF conditions. It is therefore appropriate to ask why the apparent instability of this *B*_*z*_ dip is more pronounced during the growth phase (which is made rather evident by Movie S2). Maltsev and Mingalev (2000) have computed the BI growth rate for a minimum *B*_*z*_ configuration and showed that it is proportional to the equatorial plasma *β*_*e*_ (*γ*_*BI*_ ∝ *β*_*e*_; see their equation 22). Figure S4 shows the radial profiles of *β*_*e*_ extracted from the simulation at different times in the format similar to Figures S1 and S2. Figure S4 demonstrates that over the first 40 min of the growth phase, *β*_*e*_ grows by a factor of ∼ 4–5 in the region of the *B*_*z*_ minimum which, taking into account the theoretical prediction by Maltsev and Mingalev (2000), suggests why the growth rate increases in the course of the growth phase. Furthermore, using numerical simulations Zhu et al. (2004) showed that for sufficiently thin current sheets, ≈ 1*R*_*e*_, *γ*_*BI*_ is completely suppressed for $\beta_{e}≲10$ with a sharp growth ensuing for *β*_*e*_ > 10 peaking at *β*_*e*_ ≈ 30 (see their Figure 4). In our global simulation we find qualitative agreement with these results in that with a similar current sheet thickness the onset of BI growth corresponds to *β*_*e*_ ≈ 10.

The increase in *β*_*e*_ in the course of the growth phase is chiefly the result of magnetotail stretching and magnetic flux depletion. An immediate corollary of this is that the state of the inner magnetotail, that is, the level of its stretching prior to the start of the growth phase, as well as the details of the growth phase driving (e.g., the magnitude of the electric field at the subsolar magnetopause), have important implications for the BI stability of the *B*_*z*_ dip during the growth phase and hence for the probability of observing auroral beads and their intensity. In particular, a more stretched tail prior to the start of the growth phase would imply a stronger instability and more pronounced auroral beading, other things being equal. Furthermore, given appropriate preconditioning and driving, the presence of the *B*_*z*_ dip on top of the progressive depletion of the magnetic field (Figure S2) may explain occasional—but rare—in situ observations of very low *B*_*z*_ values in this region (Ohtani & Motoba, 2017).

An implication of the above discussion is that the presence of auroral beads in the growth phase or even immediately before onset (e.g., Kalmoni et al., 2017; Nishimura et al., 2016) is not necessarily a cause of the onset, but rather an intrinsic feature of the state of the coupled inner magnetotail‐auroral ionosphere system during the growth phase. In this sense, the relationship of auroral beads to onset may be not causal, but correlative. Alternatively, Oberhagemann and Mann (2019) posit, based on theoretical considerations, that anisotropic ballooning instability can be both a source of auroral beads and a trigger of the auroral substorm onset.

Returning to the *B*_*z*_ minimum, in our simulation of a synthetic magnetospheric substorm it forms at *R*∼9–10 *R*_E_ on the night side, where *R* is the equatorial distance from Earth. The background geomagnetic field in that region is still relatively large (∼ 7 nT) even at the end of the substorm growth phase, when it is most depleted. Therefore, even when BI instability develops nonlinearly in the *B*_*z*_ minimum region, reconnection (otherwise allowed due to numerical resistivity) does not ensue. This is distinctly different from fully kinetic particle‐in‐cell (PIC) simulations (e.g., Pritchett & Coroniti, 2011, 2013) of configurations with an imposed *B*_*z*_ minimum, where reconnection is initiated in the wakes of earthward moving interchange heads due to magnetic flux depletion. Such reconnection would require *β*_*e*_∼500, which was never achieved in our simulation, where the highest *β*_*e*_ value was ∼ 100 (Figure S3). We note, however, that the equatorial *B*_*z*_ value in the vicinity of the *B*_*z*_ dip at the end of the growth phase in our simulation (Figure S2) is 1–2 *σ* (standard deviations) below the median statistical value cited by Ohtani and Motoba (2017). Therefore, the state of the simulated magnetotail is statistically representative of the state of the stretched tail in reality.

One other significant difference between the MHD and kinetic BI modes is their azimuthal propagation direction. In the present simulation, the drift of the BI heads can be either duskward (premidnight) or dawnward (postmidnight) with typical azimuthal speeds of O(10) km/s and are caused by MHD stresses. In the PIC simulations (Pritchett & Coroniti, 2011) the direction of the azimuthal drift is defined by the direction of motion of the cross‐tail current carriers in a thin current sheet (ions or electrons). Furthermore, although the scales of the observed and GAMERA‐simulated BI heads in Figure 4 are similar, there are also substantial differences in their propagation speed. Indeed, the observed azimuthal drift velocities (0.2°/s) correspond to O(100) km/s phase speed in the magnetosphere, which is close to those predicted by the PIC simulations (yielding a fraction of the ion thermal velocity). This, however, may be due to the fact that the slower‐drifting MHD BI heads reported here are more elusive for spacecraft observations. Despite the differences with the specific observations (Figure 4a) and PIC simulations, we note that the results of our global MHD simulation is well within the range of statistical properties of auroral beads, including their propagation direction (can be either dawnward or duskward), speed and size (e.g., Nishimura et al., 2016). One of the interesting avenues for future work would be to investigate the effects of the feedback of a realistic ionospheric conductance model and solar wind driving (c.f. Merkin et al., 2019).

An MHD simulation at the scale presented above, namely, resolving the ion kinetic scale within a fluid model, marks a qualitative transition in global magnetosphere modeling. Going beyond these scales requires departing from ideal MHD. Describing electron‐scale reconnection physics may ultimately be achieved by locally embedding fully kinetic codes within global fluid models (Chen et al., 2017). At the same time, capturing larger‐scale ion kinetic processes, for example, the kinetic BI effects mentioned above or thin current sheets (e.g., Sergeev, V., Angelopoulos, V., et al. 2011), requires developing global hybrid approaches (e.g., Lin et al., 2017; Palmroth et al., 2018) or otherwise incorporating feedback of ion kinetics in fluid models (e.g., Mignone et al., 2018). However, it is imperative that any attempt to augment a fluid model with kinetic effects first ensure that global and mesoscale fluid physics are properly captured and described all the way down to the kinetic scale. Having now demonstrated that our GAMERA model satisfies this requirement, we are confident that future work concentrating on incorporation into it of kinetic effects will be put on a firm footing.

## Supporting information



Supporting Information S1

Movie S1

Movie S2

## Data Availability

Data used to create the figures presented are archived and available online (via https://doi.org/10.5281/zenodo.3835863). In addition to the postprocessed data used to generate figures stored on Zenodo, raw simulation data are preserved on the Cheyenne supercomputer and can be made available upon request.
