# Supplementary material for: Ballooning‐Interchange Instability in the Near‐Earth Plasma Sheet and Auroral Beads: Global Magnetospheric Modeling at the Limit of the MHD Approximation
Source: Geophys Res Lett. 2020 Jul 14;47(14):e2020GL088227. doi: 10.1029/2020GL088227 (PMC7507213; doi:10.1029/2020GL088227)
Supplement: Supplementary file 1 — Supporting Information S1 [file GRL-47-e2020GL088227-s001.pdf]

Ballooning-interchange instability in the near-Earth plasma sheet and auroral beads: Global magnetospheric modeling at the limit of the MHD approximation

K. A. Sorathia[1], V.G. Merkin[1], E.V. Panov[2], B. Zhang[3], J.G. Lyon[4], J. Garretson[1], A.Y. Ukhorskiy[1], S. Ohtani[1], M. Sitnov[1], M. Wiltberger[5]

[1]The Johns Hopkins University Applied Physics Laboratory, Laurel, MD, USA

[2]Space Research Institute, Austrian Academy of Sciences, Graz, Austria

[3]Department of Earth Sciences, University of Hong Kong, Hong Kong

[4]Department of Physics and Astronomy, Dartmouth College, Hanover, NH, USA

[5]High Altitude Observatory, National Center for Atmospheric Research, Boulder, CO, USA

Contents of this file

1. Figures S1 to S4

Additional Supporting Information (Files uploaded separately)

1. Captions for Movies S1 to S2

Movie S1. Simulation at a glance. Left panel) Residual, i.e. non-dipolar, vertical magnetic field in the SM equator. The $B_Z = 0$ contour is overlaid in magenta. Time is measured relative to the southward IMF turn ($T = 0$). Right panel) Gas pressure in

the meridional plane. Insets show field-aligned currents on the ionospheric grid in the northern and southern hemisphere.

Movie S2. Magnetosphere-ionosphere coupling during growth phase. Top panel) Field-aligned current density dipole-mapped from the inner boundary of the MHD simulation, at $2R_E$, to the ionospheric grid. Grid denotes lines of constant magnetic latitude and longitude. Bottom panel) Flux-tube integrated entropy calculated at each point in the equator. Overlaid contours are the magnetic field projection of the regular MLON-MLAT grid shown in the top panel to the magnetospheric equator.

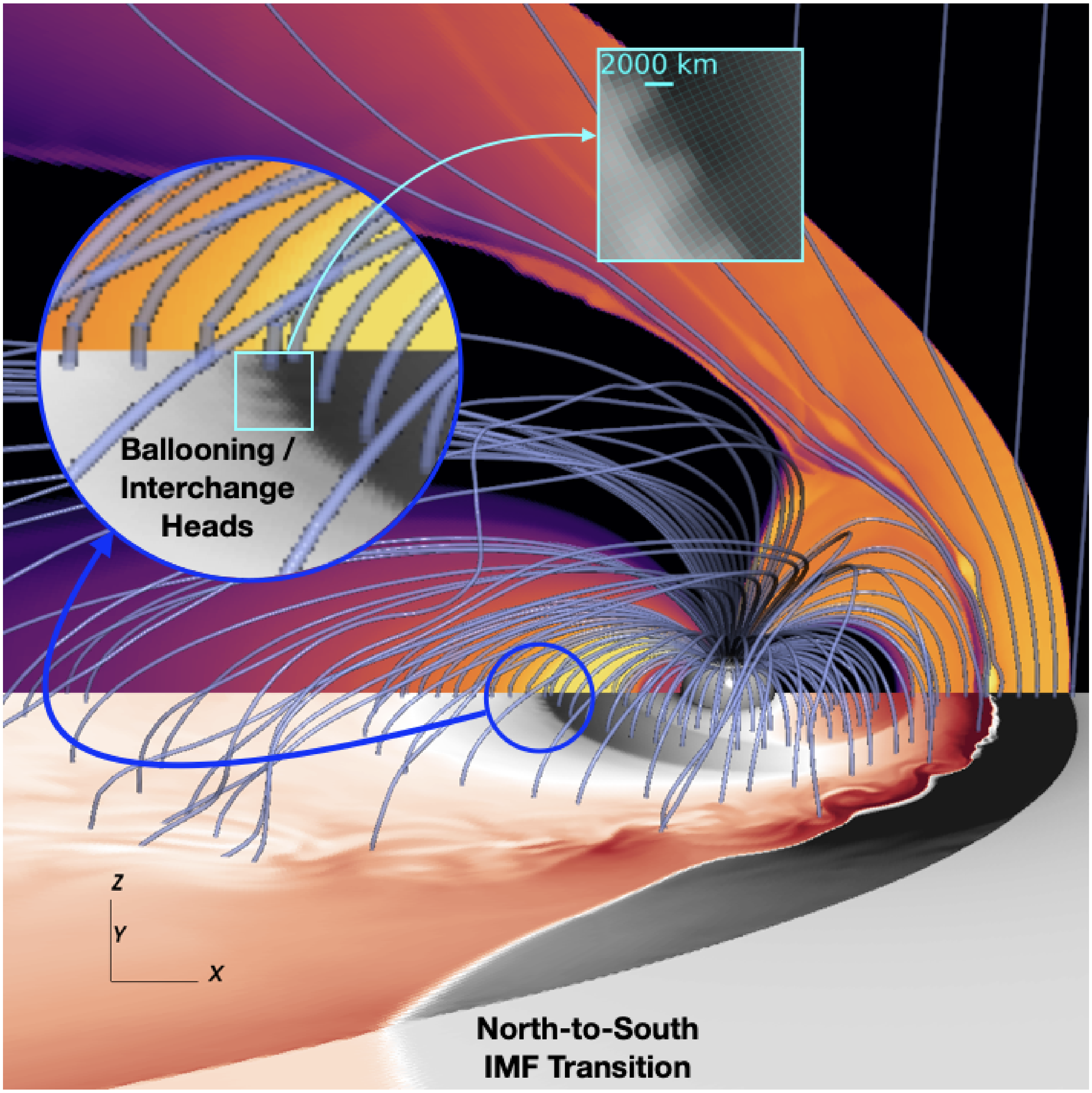

Figure S1. Three-dimensional visualization of the global simulated magnetosphere during growth phase. Depicted in the main panel are equatorial residual magnetic field and meridional pressure along with a sample of magnetic field lines. Progressive zoom-ins illustrate the BI structures with the deepest layer of zoom also showing the equatorial computational grid.

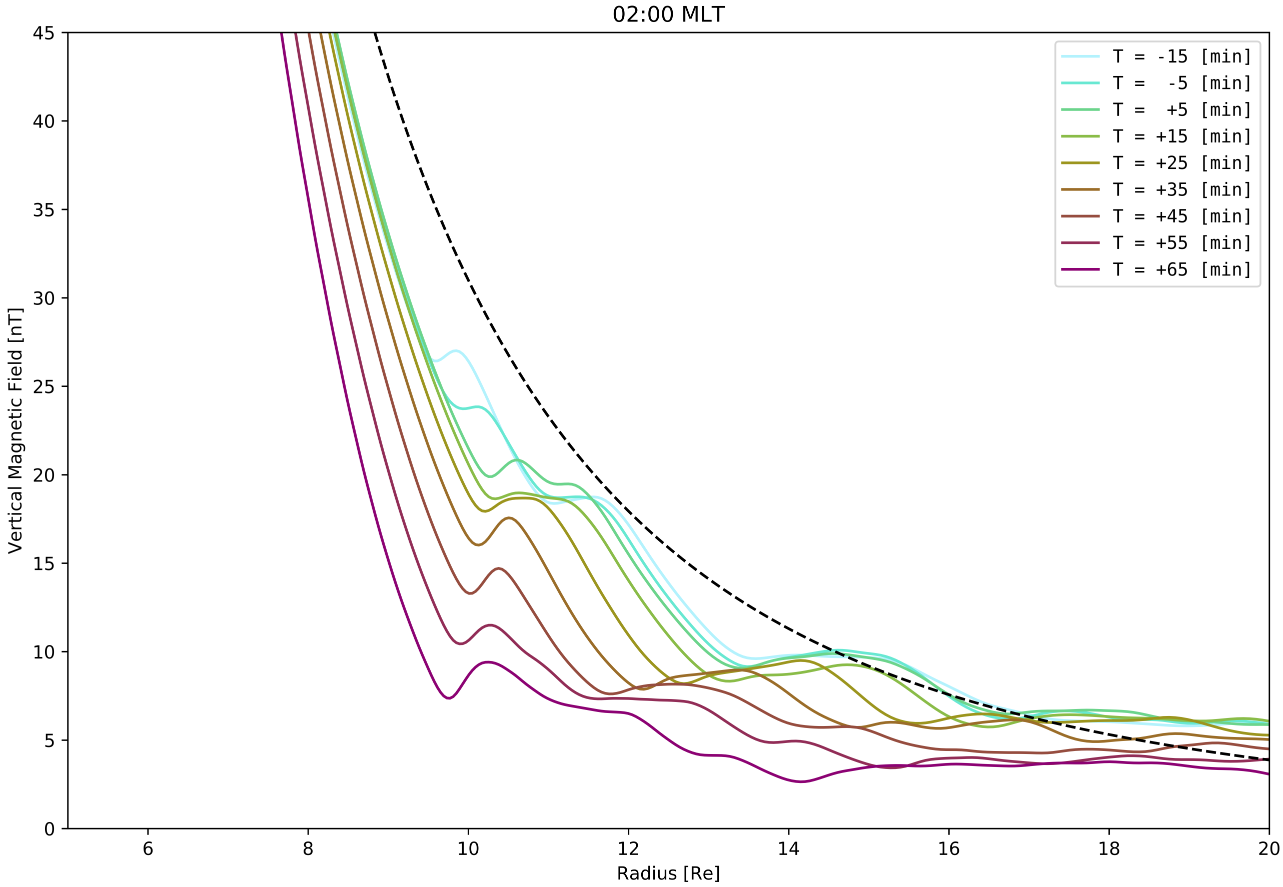

Figure S2. Radial profiles, taken in the equator at 0200 MLT, of vertical magnetic field at selected times. Time is measured relative to southward IMF turning ($T = 0$). Dashed line shows the corresponding dipole field profile.

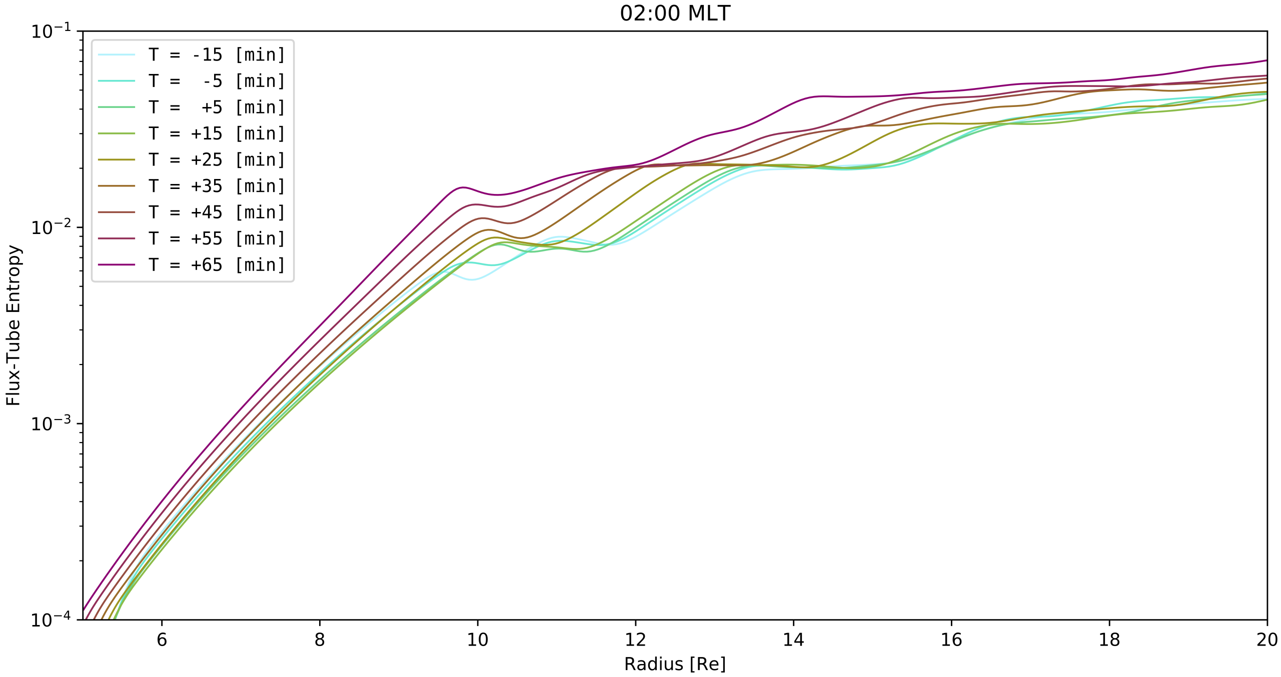

Figure S3. Flux-tube entropy plotted in the same manner as Fig. S1.

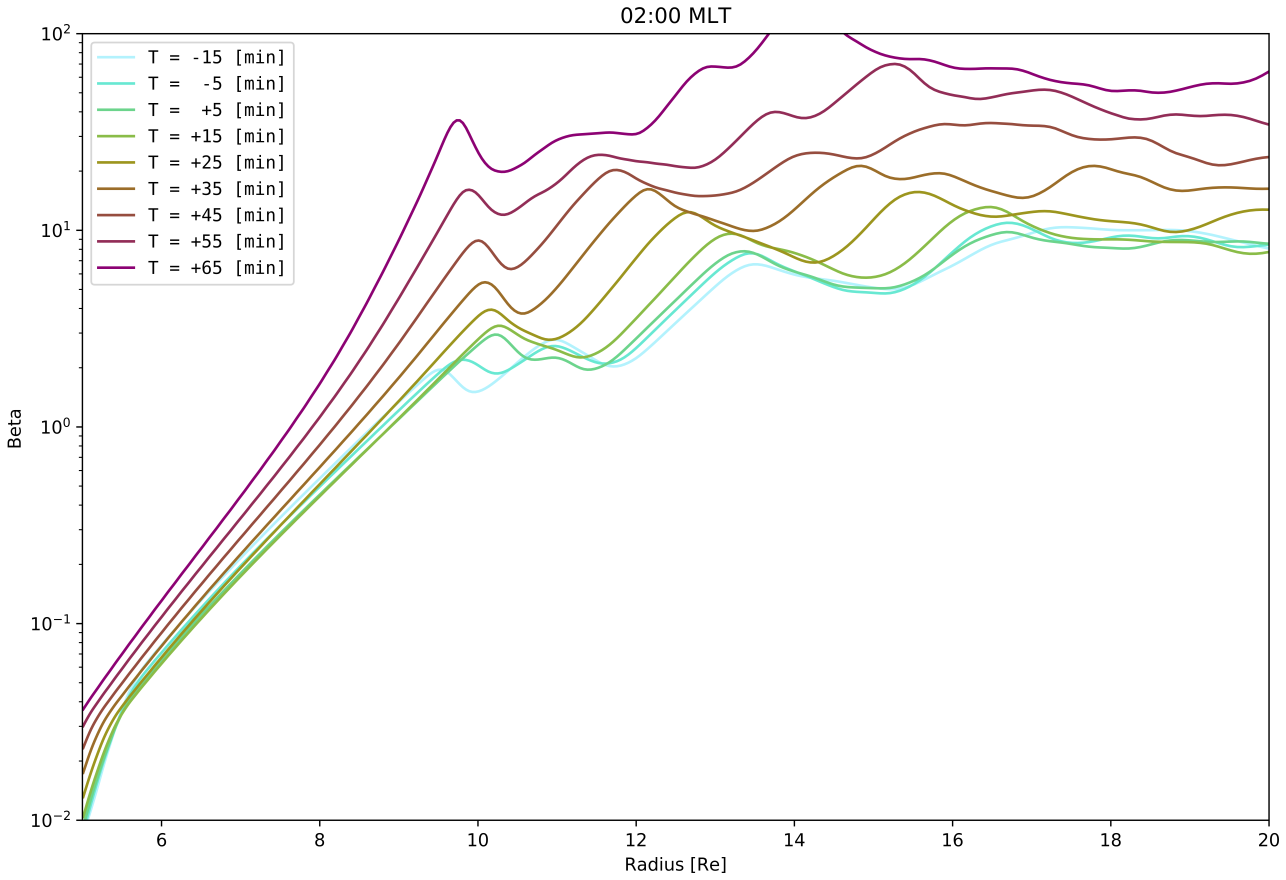

Figure S4. Plasma β plotted in the same manner as Fig. S1.
